# Physiological and Health-Related Adaptations to Low-Volume Interval Training: Influences of Nutrition and Sex

**DOI:** 10.1007/s40279-014-0259-6

**Published:** 2014-10-30

**Authors:** Martin J. Gibala, Jenna B. Gillen, Michael E. Percival

**Affiliations:** Department of Kinesiology, McMaster University, Hamilton, ON L8S 4K1 Canada

## Abstract

Interval training refers to the basic concept of alternating periods of relatively intense exercise with periods of lower-intensity effort or complete rest for recovery. Low-volume interval training refers to sessions that involve a relatively small total amount of exercise (i.e. ≤10 min of intense exercise), compared with traditional moderate-intensity continuous training (MICT) protocols that are generally reflected in public health guidelines. In an effort to standardize terminology, a classification scheme was recently proposed in which the term ‘high-intensity interval training’ (HIIT) be used to describe protocols in which the training stimulus is ‘near maximal’ or the target intensity is between 80 and 100 % of maximal heart rate, and ‘sprint interval training’ (SIT) be used for protocols that involve ‘all out’ or ‘supramaximal’ efforts, in which target intensities correspond to workloads greater than what is required to elicit 100 % of maximal oxygen uptake (*V*O_2max_). Both low-volume SIT and HIIT constitute relatively time-efficient training strategies to rapidly enhance the capacity for aerobic energy metabolism and elicit physiological remodeling that resembles changes normally associated with high-volume MICT. Short-term SIT and HIIT protocols have also been shown to improve health-related indices, including cardiorespiratory fitness and markers of glycemic control in both healthy individuals and those at risk for, or afflicted by, cardiometabolic diseases. Recent evidence from a limited number of studies has highlighted potential sex-based differences in the adaptive response to SIT in particular. It has also been suggested that specific nutritional interventions, in particular those that can augment muscle buffering capacity, such as sodium bicarbonate, may enhance the adaptive response to low-volume interval training.

## Introduction

Interval training has long been considered an essential component of programs designed to maximize performance in highly-trained athletes, which typically involve a relatively high volume of submaximal, moderate-intensity continuous training (MICT) [1–5]. While less well-appreciated, interval training per se is a potent stimulus to induce physiological remodeling that resembles—or indeed may be superior to—changes typically associated with traditional endurance training [6–9]. This brief commentary focuses on physiological and health-related adaptations to low-volume interval training, which is characterized by sessions that involve a relatively small total amount of exercise, compared with MICT protocols that are generally reflected in current public health guidelines [10–12]. The present work builds upon recent reviews by some of the same authors [6, 7, 13] and also considers possible sex-based differences in the adaptive response to this type of training, in addition to the potential influence of nutritional manipulation on training outcomes. For a more comprehensive analyses of the physiological and health adaptations to interval training—and in particular comparisons between relatively high-volume interval training and traditional MICT, in which efforts are made to match energy expenditure—the reader is referred to recent reviews by others, including work that has focused on those at risk for, or afflicted by, cardiometabolic disorders such as cardiovascular disease and type 2 diabetes [8, 14–18]. Several other recent reviews have considered in detail various aspects of program design, with a particular focus on the application of interval training for athletic performance [19, 20].

## Characterizing the Training Stimulus: Standardizing Terminology

Interval training refers to the basic concept of alternating periods of relatively intense exercise with periods of lower-intensity effort or complete rest for recovery. A wide range of terms have been used by different groups to describe various interval training protocols, which has led to a dizzying array of acronyms and general lack of standardization in the literature. Weston et al. [8] recently proposed a simple classification scheme for interval training based on exercise intensity as part of an effort to standardize terminology in future studies. The authors suggested that the term ‘high intensity interval training’ (HIIT) be used to describe protocols in which the training stimulus is ‘near maximal’ or the target intensity is between 80 and 100 % of maximal heart rate (HR_max_). In contrast, the authors advocated use of the term ‘sprint interval training’ (SIT) for protocols that involve ‘all out’ or ‘supramaximal’ efforts, in which target intensities correspond to workloads greater than what is required to elicit 100 % of maximal oxygen uptake (*V*O_2max_). Weston et al. [8] also suggested that the standardized term ‘moderate-intensity continuous training’ be used where appropriate in comparative studies. Other authors [21] have also recently considered various methodological approaches for the classification of interval training, including the use of turn-point or threshold models to prescribe intensity rather than percentages of HR_max_ or *V*O_2max_.

**Fig. 1:**
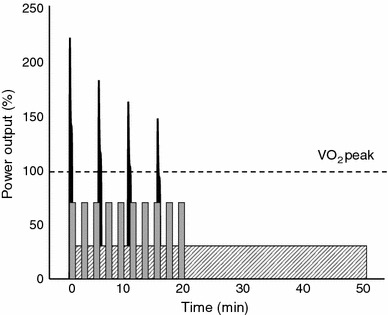
Examples of protocols employed in interval training studies, expressed relative to PPO that is required to elicit *V*O_2max_ or *V*O_2__peak_. The figure shows typical MICT, e.g. 50 min at ~35 % of PPO, which elicits ~70 % of HR_max_ (*hatched box*); low-volume HIIT, e.g. 10 × 1 min at a constant workload corresponding to ~75 % of PPO, interspersed with 1 min of recovery, which elicits ~85–90 % of HR_max_ during the intervals (*grey bars*); and low-volume SIT, e.g. 4 × 30 s ‘all out’ effort at a variable power output corresponding to ~175 % of PPO (averaged over the course of the intervals), interspersed with 4 min of recovery, which elicits ~90–95 % of HR_max_ during the intervals (*black bars*). Power output and heart rate estimates are derived from Little et al. [31] and Skelly et al. [60]. *PPO*, peak power output, *VO*_*2max*_, maximal oxygen uptake, *VO*_*2*_ *peak*, peak *VO*_2_, *MICT*, moderate-intensity continuous exercise, *HR*_*max*_, maximum heart rate, *HIIT*, high-intensity interval training, *SIT*, sprint-interval training

We applaud the efforts to try and standardize interval training terminology research in future studies. While cognizant of the potential value in other approaches [21, 22], especially for training prescription for athletes, we will employ the basic classification scheme proposed by Weston et al. [8] in the present review, given the widespread use of percentages of HR_max_ and *V*O_2max_ to describe relative exercise intensity. Weston et al. [8] used the specific descriptors ‘peak heart rate’ and ‘maximal oxygen uptake’; in the present review we will use ‘HR_max_’ and ‘*V*O_2max_’ to describe relative intensities scaled to ‘peak’ and/or ‘maximal’ heart rate and oxygen uptake, respectively, for simplicity and consistency, and regardless of the specific term used in original studies that are cited here. There is no universal definition of what constitutes ‘low volume’ interval training, but in the present review we will consider protocols in which the total amount of intense exercise performed during a training session was ≤10 min within a training session, i.e. the summed total duration of the hard efforts, excluding the recovery periods and any warm-up or cool-down. Based on this depiction and the classification scheme proposed by Weston et al. [8], an example of a low-volume HIIT protocol is ten 60-s cycling efforts at an intensity that elicits ~85–90 % HR_max_, interspersed by 60 s of recovery [23]. An example of low-volume SIT is the repeated Wingate Test model, which typically consists of four to six 30-s all-out efforts at mean power outputs corresponding to ~250 % of the absolute workload elicited at the end of an incremental *V*O_2max_, interspersed with a few minutes of recovery [24]. An overview of common protocols employed in interval training studies is depicted in Fig. 1.

## Physiological Adaptations to Low-Volume Interval Training

It has been recognized for some time that relatively short-term SIT and HIIT protocols can rapidly enhance the capacity for aerobic energy metabolism [25, 26] and elicit physiological remodeling that resembles changes induced by MICT. While relatively few direct comparisons have been made, in one of the first studies to compare skeletal muscle adaptations after low-volume interval versus traditional endurance training, Gibala et al. [27] reported increases in various markers of mitochondrial content after only six sessions of SIT or MICT over 2 weeks. Young active men performed either four to six Wingate Tests with 4 min of recovery in between, or 90–120 min of continuous cycling at approximately 65 % *V*O_2max_, per session. Total training time commitment was ~2.5 h and ~10.5 h for SIT and MICT, respectively, and total training volume based on mechanical work was ~ 90% lower in the SIT group (~630 vs. ~6,500 kJ). Skeletal muscle needle biopsy samples obtained before and after training revealed similar increases in the maximal activities of citrate synthase and cytochrome c oxidase (COX) and the protein content of COX subunits II and IV, with no difference between groups [27]. Similar adaptations have been reported in studies that examined skeletal muscle adaptations to 2-week HIIT protocols [23, 28]. Burgomaster et al. [29] compared two groups of previously sedentary individuals who performed either 6 weeks of Wingate-based SIT, 3 days/week similar to Gibala et al. [27], or MICT that involved 40–60 min of continuous cycling at 65 % of *V*O_2peak_, 5 days/week. Both protocols elicited similar increases in the maximal activities of mitochondrial markers, including citrate synthase, pyruvate dehydrogenase and 3-hydroxyacyl CoA dehydrogenase, and also reduced muscle glycogen and phosphocreatine utilization during submaximal, matched-work exercise following training in both groups.

The molecular mechanisms underlying skeletal muscle metabolic adaptations to low-volume interval training have been reviewed elsewhere [6], but in general many of the underlying signaling events appear to be at least qualitatively similar to processes proposed to regulate adaptations to MICT [31]. For example, similar acute activation of signaling pathways involved in mitochondrial biogenesis have been reported after a single session of SIT, HIIT, or MICT, including phosphorylation of 5′AMP (adenosine monophosphate)-activated protein kinase (AMPK), p38 mitogen-activated protein kinase (p38 MAPK), and p53 [31, 32]. This suggests that at least some of the underlying mechanisms are qualitatively similar between the two training modalities, although it is also possible that interval training may stimulate pathways that initially differ from MICT but ultimately converge to elicit specific adaptive responses, e.g. mitochondrial biogenesis [3].

With respect to cardiovascular adaptations in healthy individuals, Rakobowchuk et al. [33] reported, in a companion paper that was based on the study by Burgomaster et al. [30], similar improvements in peripheral vascular structure and function, including popliteal artery distensibility and flow-mediated dilation, after 6 weeks of low-volume SIT and MICT. A subsequent study from another laboratory that employed the same experimental design [34] found that 6 weeks of SIT and MICT were equally effective in increasing skeletal muscle microvascular density and enzyme content, despite large differences in total training volume. Consistent with these findings, McKay et al. [35] reported that eight sessions of either SIT (eight to twelve 60-s intervals at 120 % *V*O_2max_, separated by 60 s of rest) or MICT (90–120 min at 65 % *V*O_2max_) improved muscle O_2_ utilization kinetics, suggestive of adaptations in local microvascular perfusion. Bailey et al. [36] also found that six sessions of Wingate-based SIT over 2 weeks accelerated muscle O_2_ utilization kinetics; however, no improvement was seen after MICT. With regards to central adaptations, MacPherson et al. [37] found that cardiac output, based on an acetylene non-rebreathing technique, was increased after 6 weeks of MICT but not SIT, despite similar improvements in *V*O_2max_. The authors suggested the early time course of cardiovascular adaptation to SIT and MICT may differ, with peripheral factors (i.e. enhanced O_2_ extraction) being more important for the former. In support of this interpretation, Jacobs et al. [38] showed that 2 weeks of SIT increased *V*O_2max_ and skeletal muscle respiratory capacity, but not cardiac output based on a nitrous oxide rebreathing method. In contrast, Esfandiari et al. [39] recently reported that Doppler-derived measures of end-diastolic volume, stroke volume, and cardiac output, as well as blood volume and *V*O_2max_, were increased to a similar extent after a 2-week HIT protocol that was modeled after Little et al. [29], as well as a high-volume MICT protocol. While additional work is warranted to clarify the precise nature and time course of the mechanisms involved, a substantive body of evidence suggests that low-volume SIT and HIIT protocols constitute relatively time-efficient training strategies to induce an array of physiological adaptations that resemble changes normally associated with high-volume MICT, at least over the short-term, i.e. up to several weeks.

## Changes in Health-Related Indices After Low-Volume Interval Training

Cardiorespiratory fitness has been documented to be a stronger predictor of risk for adverse health outcomes than traditional risk factors such as hypertension, smoking, obesity, and hyperlipidemia [40]. As little as six sessions of low-volume SIT over 2 weeks has been shown to improve cardiorespiratory fitness, as reflected by increased *V*O_2max_ during an incremental exercise test to exhaustion [36, 41–43]. A recent systematic review and meta-analysis based on 16 studies summarized the improvements in cardiorespiratory fitness following SIT in young healthy individuals, and reported a moderate to large effect size in comparison to non-exercise control groups, and no difference when compared with MICT [44]. Interestingly, the effect of SIT on aerobic capacity was unaffected by initial fitness level (sedentary, recreational, trained), length of training intervention (<6 weeks, ≥6 weeks), or mode of training (cycling, running, rowing). The aggregate increase in *V*O_2max_ after SIT was 3.6 ml/kg/min (8 %), an improvement that would approximately translate into a 15 and 19 % lower risk of all-cause and cardiovascular disease mortality, respectively [45]. Similar to the findings of Gist et al. [44], another meta-analysis of 19 studies by Sloth and colleagues [46] reported that *V*O_2max_ increased by a range of 4–13 % after 2–8 weeks of SIT in healthy sedentary or recreationally active adults. Data from individual studies, reminiscent of classic work by Tabata et al. [27], reveal that a surprisingly small total SIT dose can elicit relatively large changes in *V*O_2max_. For example, Ma et al. [47] recently showed that 16 sessions of a protocol that involved eight 20-s cycling efforts at 170 % *V*O_2max_, interspersed with 10 s recovery, improved *V*O_2max_ in young men by 19 % after 4 weeks. Similar improvements have also been reported using protocols involving 10 s [41] and 20 s all-out cycling sprints [48]. There are less data on the effects of low-volume HIIT but recent studies showed improvements in *V*O_2max_ in overweight women after 6 weeks of training [49], and in patients with coronary artery disease after 12 weeks of training [50], with the latter change being comparable to a similar period of MICT that involved twice as much exercise. Relative intensity appears to be important when it comes to HIIT protocols, with a recent study showing that cardiovascular adaptation, as reflected by change in *V*O_2max_, was reduced when overweight/obese men trained using a 10 × 60-s protocol at an intensity equivalent to 70 % of peak power elicited at *V*O_2max_ compared with 100 % of peak power [51].

A few studies have reported improvements in glycemic control after short-term, low-volume interval training, in both healthy individuals and those at risk or afflicted by cardiometabolic diseases. Babraj et al. [52] were the first to report that six sessions of SIT over 2 weeks was a sufficient stimulus to improve insulin sensitivity in young active men, measured using oral glucose tolerance tests (OGTT). These findings were confirmed by Richards et al. [53], who showed that, using the hyperinsulinemic euglycemic clamp technique, insulin sensitivity was improved 72 h following a 2-week SIT intervention in healthy men and women. Whyte et al. [43] reported 2 weeks of SIT in overweight but otherwise healthy adults improved OGTT-derived estimates of insulin sensitivity when measured 24 but not 72 h after training, and Hood et al. [23] showed that six sessions of HIIT over 2 weeks improved insulin sensitivity based on fasting measures of glucose and insulin in previously sedentary individuals. Using continuous glucose monitoring (CGM), Little et al. [54] reported lower 24-h blood glucose concentration in patients with type 2 diabetes when measured 72 h following a 2-week HIIT protocol. All of these studies have been relatively short-term investigations on small numbers of subjects, without direct comparison with high-volume MICT protocols.

Recent evidence has also highlighted the potential for low-volume interval training to induce favorable changes in body composition. For example, 18 sessions of all-out running SIT over 6 weeks decreased whole body fat mass and increased whole body fat-free mass in recreationally active men [55] and women [56]. A cycling-based SIT protocol involving 60 repetitions of 8 s all-out sprints, interspersed with 12 s recovery, performed 3×/week for 15 weeks, was also shown to be more effective than an MICT protocol involving 40 min of cycling at 60 % of *V*O_2max_ for decreasing whole body and abdominal fat mass in women [57]. Gillen et al. [49] also reported reductions in whole body and abdominal fat mass following 6 weeks of low-volume HIIT in overweight women. Boutcher [58] has discussed the various factors that could mediate changes in body composition after low-volume interval training, and some evidence has been presented in support of specific mechanisms, including increased post-exercise oxygen consumption or changes in appetite [59–61].

## Potential Sex-Based Differences in the Adaptive Response to Low-Volume Interval Training

Recent evidence from a limited number of studies has highlighted potential sex-based differences in the adaptive response to SIT. Metcalfe et al. [48] utilized a protocol that involved two 20-s all-out sprints within a 10-min training session that otherwise consisted of low-intensity cycling, including warm-up and cool-down. When previously sedentary but otherwise healthy men and women trained 3×/week for 6 weeks, gains in aerobic capacity were similar, but insulin sensitivity measured using OGTTs was improved in men only. We also previously observed no change in OGTT-derived estimates of insulin sensitivity after a 6-week HIIT intervention in women [49], although this study did not involve a sex-based comparison. It has been suggested that high rates of glycogen breakdown and subsequent resynthesis following intense exercise may explain the rapid improvement in insulin sensitivity after SIT [48]. However, in comparison to men, women are reported to break down 42 % less muscle glycogen in type 1 fibers during a single Wingate sprint [62], which is supported by a lower blood lactate accumulation following single [63, 64] and repeated 30-s sprints [64]. The reduced rate of glycogenolysis may be associated with lower basal activities of muscle phosphofructokinase [65] and lactate dehydrogenase reported in women [65, 66], or a lower catecholamine response to repeated sprints [63, 64]. Interestingly, women are suggested to have a greater pre-disposition for aerobic metabolism as estimates based on respiratory gas analyses suggest that the aerobic contribution to a 30-s sprint is 25 % higher in women compared with men [67]. This difference could also explain in part the relative glycogen sparing that has been observed in women [62], with potential effects of associated metabolic byproducts on downstream signaling events that regulate muscle adaptations. Divergent adaptations in skeletal muscle remodeling were reported following a 3-week SIT intervention in active young men and women. Oral administration of deuterium oxide revealed higher rates of muscle protein synthesis in men over the course of training in both the mixed and cytosolic fractions [68]. However, it is important to note that other studies involving mixed cohorts of men and women have not described sex-based differences in the adaptive response to interval training [23, 29, 53], although these studies were not specifically designed to address this issue. Clearly, additional well-controlled studies are warranted to determine whether women might in fact ‘respond less’ to low-volume SIT, using best practice designs that control for various factors, such as menstrual cycle phase and relative fitness, that can increase variance and lead to false conclusions regarding potential sex differences [69].

## Can Nutrition Alter the Adaptive Response to Low-Volume Interval Training?

Based on our understanding of the molecular biology underlying muscle adaptation to exercise training [31], and the potential for nutrition to modulate training adaptation [70], interventions in theory could augment the adaptive response to interval training by: (1) improving energy metabolism during exercise, which could facilitate greater total work and an enhanced chronic training stimulus; or (2) promoting some aspect of the adaptive response during recovery, which could lead to enhanced physiological adaptations over time [71]. A large number of potential candidates, including, for example, caffeine and creatine, have been identified and discussed in detail elsewhere [72–74]. For the purposes of this review, only a few specific nutritional practices or supplements will be considered here.

Arguably the best evidence to date regarding the potential for nutritional manipulation to enhance physiological adaptation to interval training is research on carbohydrate (CHO) restriction protocols [75]. As originally proposed by Hansen et al. [76], the basic concept is that training in a CHO-restricted state and/or with reduced glycogen availability could serve to augment the acute molecular signaling response to exercise [76]. A typical research design involves a comparison of two groups who either train once daily, or twice a day, every other day, for up to several weeks, with the manipulation resulting in the latter performing approximately half of all training sessions in a ‘reduced’ state [77–79]. These studies have generally failed to show any beneficial effect on performance, at least using whole-body exercise protocols that resemble normal athletic competition, and indeed perception of effort may be increased. However, from a basic science standpoint, studies have shown that CHO-restricted training can enhance mitochondrial adaptation, even in highly trained individuals. This could result from the transient yet repeated enhancement of acute signaling proteins that regulate mitochondrial biogenesis. For example, several studies have shown that the acute activation of AMPK [80], p38-MAPK [81], and p53 [82] is amplified when interval training is commenced with low glycogen per se or reduced CHO availability. Specifically in regards to AMPK regulation, the enhanced signaling may be mediated through a greater liberation of the regulatory beta subunit, which is sequestered under normal glycogen conditions [83–85].

Interventions that alter buffering capacity may also be of particular relevance to interval training. Repeated sprints have the potential to depress intramuscular pH to 6.5–6.8 [86–89], which may reduce glycolytic flux [87, 88] and alter calcium sensitivity/handling [90, 91]. However, the extent to which pH may play a role in fatigue has been questioned [89, 92, 93]. Nonetheless, elevated buffering capacity has been associated with enhanced sprint ability [94] and short duration endurance cycling performance [95]. Hence, research has focused on supplementing the natural buffering system in an attempt to augment physiological adaptations and performance. One method of manipulating the extracellular buffering capacity is through ingestion of sodium bicarbonate (NaHCO_3_), as the associated elevation of blood bicarbonate, pH and base excess has been proposed to elevate H^+^ efflux out of contracting muscles [96, 97]. Another hypothesis is that alkalosis may help maintain muscle membrane excitability through improved strong ion regulation [98–100]. Regardless of the potential underlying mechanism, there is good evidence to support the practice of acute NaHCO_3_ ingestion, with a recent meta-analysis finding that a dose of 0.3–0.5 g/kg body mass improved mean power output by 1.7 % (±2.0 %) during short high-intensity exercise [101]. However, it must be emphasized that not all studies have found performance improvements after NaHCO_3_ ingestion, and individual responses are variable [102].

With respect to the evidence that repeatedly induced metabolic alkalosis can augment adaptations over the course of chronic training [103–105], Edge et al. [103] reported that recreationally active women who consumed NaHCO_3_ for 8 weeks in conjunction with interval training experienced lower H^+^ accumulation during each session, and this was associated with a training-induced improvement in endurance capacity and lactate threshold. To garner insight into the potential mechanistic basis for the observed performance enhancement, Bishop and colleagues [105] had rats perform interval training five times/week for 5 weeks while supplemented with NaHCO_3_ or a placebo. Both groups increased endurance capacity, as evidenced by significant increases in running time to exhaustion in comparison to unexercised control rats. However, the rats supplemented with NaHCO_3_ showed a superior performance improvement, attributed to greater improvements in both mitochondrial mass and respiration [104]. It is important to note that within both studies, groups were matched for total work performed during each training session, and thus the enhanced muscle adaptations and performance cannot be attributed to differences in total training volume.

**Fig. 2:**
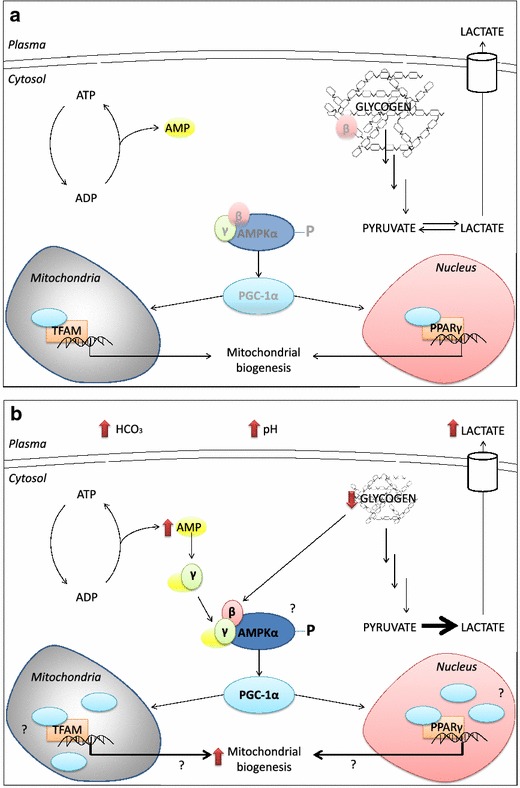
Hypothesized acute signaling mechanism underpinning enhanced training adaptations in response to chronic NaHCO_3_ supplementation. **a** AMPK acts as an energy sensor that is activated by various signals generated during muscle contraction (e.g. increased AMP), which subsequently activates PGC-1α, leading to increased transcription of various genes involved in mitochondrial biogenesis. **b** Supplementation with NaHCO_3_ may alter muscle metabolism, resulting in greater increases in AMP, which could enhance AMPK activation through interaction with the γ-subunit. Glycogen utilization during exercise is also increased after NaHCO_3_ supplementation, and the AMPK β-subunit that is sequestered by glycogen may be liberated to a greater extent. Greater AMPK activation could result in greater downstream signaling, including activation of PGC-1α and increased gene expression. *Red arrows* depict potential mechanisms that are supported by experimental data, whereas *question marks* indicate areas that remain to be directly investigated. *NaHCO*_*3*_ sodium bicarbonate, *AMPK* adenosine monophosphate kinase, *PGC-1α* peroxisome proliferator-activated receptor *γ* co-activator

Several studies have investigated the effects of NaHCO_3_ on muscle metabolism during acute exercise and these provide additional insight into potential cellular mechanisms involved [96, 97, 106–108]. A relatively common finding is that NaHCO_3_ supplementation increases the rate of muscle glycogen degradation [96, 97], and this is associated with increased content of lactate in muscle and/or blood [96, 97, 106, 107]. Hollidge-Horvat et al. [96] also reported that exercise following NaHCO_3_ supplementation resulted in elevated muscle content of adenosine diphosphate, adenosine monophosphate, and inorganic phosphate, while phosphocreatine content was reduced in comparison to placebo. The authors proposed that altered cellular energetics were due to higher glycogen utilization, facilitated by a lack of inhibition on glycolytic flux, which conversely reduced free fatty acid (FFA) metabolism [96]. In support of this interpretation, the authors also found a marked decline in free carnitine as well as muscle pH following supplementation. Both mechanisms have been implicated in the inhibition of carnitine palmitoyltransferase 1 (CPT-1), the rate-limiting enzyme of long-chain fatty acid transport into mitochondria for beta oxidation [109–112]. Hollidge-Horvat and colleagues further suggested that decreased FFA utilization would cause a decline in the ratio of reduced/oxidized nicotinamide adenine dinucletides in the mitochondria, requiring the observed alterations in cellular energetics to drive oxidative phosphorylation [96]. The hypothesized acute signaling mechanism underpinning enhanced training adaptations in response to chronic NaHCO_3_ supplementation is depicted in Fig. 2.

It has also been speculated that nutritional manipulation of intramuscular buffering capacity could augment adaptations to interval training. The dipeptide carnosine is an important intramuscular buffer, and the intramuscular content can be increased through chronic oral supplementation with the rate-limiting amino acid precursor, β-alanine [113]. A recent meta-analysis suggests that β-alanine has ergogenic effects on exercise lasting 1–4 min with an improvement of 2.85 % in performance measures [114]. While the majority of studies have focused on performance, recent evidence suggests metabolism within the contracting muscle may be altered by β-alanine supplementation. For example Gross et al. [115] found that in response to a single, high-intensity, fixed-power, fixed-duration test, supplementation with β-alanine appeared to reduce the oxygen deficit and accumulation of both blood and muscle lactate. The authors suggested that an enhancement in oxidative phosphorylation coinciding with a decrease in substrate level phosphorylation was evident [115]. Interestingly, these results appear to contrast those found when manipulating the extracellular buffer with NaHCO_3_ supplementation, as discussed above. Aside from this initial work by Gross and colleagues, very little is known regarding other acute metabolic effects that may influence muscle signaling [115]. Recent investigations using relatively short training blocks lasting up to several weeks suggest that chronic β-alanine supplementation does not further enhance the adaptive response [115–117].

In summary, while some evidence suggests that specific nutritional interventions may alter the adaptive response to interval training, future work should focus on elucidating the underlying mechanisms as well as understanding how these may translate to increased performance during athletic competition.

## Conclusions

The majority of low-volume interval training studies have utilized relatively short intervention periods (i.e. lasting up to several weeks) and future work involving long-term (i.e. months to years) interventions is needed to advance our basic understanding of how manipulating the exercise stimulus translates into physiological remodeling. From an applied perspective, there is value in trying to establish the minimum ‘dose’ of HIIT or SIT needed to stimulate meaningful improvements in clinical markers that are associated with disease risk. This is particularly germane given that ‘lack of time’ remains the most commonly cited barrier to regular exercise participation [118], and considering evidence that suggests that low-volume interval training is perceived to be more enjoyable than MICT [119]. There is also evidence that nutritional interventions can influence both acute and chronic adaptations to interval training [74], findings that may have relevance beyond athletic performance and competitive sport. As suggested by van Loon and Tipton [120], greater adaptation efficiency has clinical relevance, especially for individuals with severe exercise intolerance.
